# Some Urea Derivatives Positively Affect Adventitious Root Formation: Old Concepts and the State of the Art

**DOI:** 10.3390/plants9030321

**Published:** 2020-03-04

**Authors:** Ricci Ada, Rolli Enrico

**Affiliations:** Dipartimento di Scienze Chimiche, della Vita e della Sostenibilità Ambientale, Università di Parma, Parco Area delle Scienze 11/A, 43124 Parma, Italy; enrico.rolli@unipr.it

**Keywords:** adventitious rooting adjuvants, auxin, urea derivatives

## Abstract

The success of vegetative propagation programmes strongly depends on adventitious rooting, a postembryonic developmental process whereby new roots can be induced from differentiated cells in positions where normally they do not arise. This auxin-dependent organogenesis has been studied at molecular, cellular, and developmental levels, and our knowledge of the process has improved in recent years. However, bioactive compounds that enhance adventitious root formation and possibly reduce undesirable auxinic side effects are still needed to ameliorate this process. From this point of view, our structure–activity relationship studies concerning urea derivatives revealed that some of them, more specifically, the *N*,*N*′-bis-(2,3-methylenedioxyphenyl)urea (2,3-MDPU), the *N*,*N*′-bis-(3,4-methylenedioxyphenyl)urea (3,4-MDPU), the 1,3-di(benzo[*d*]oxazol-5-yl)urea (5-BDPU), and the 1,3-di(benzo[*d*]oxazol-6-yl)urea (6-BDPU), constitute a category of adventitious rooting adjuvants. The results of our studies are presented here, in order either to highlight the positive effects of the supplementation of these urea derivatives, or to better understand the nature of their interaction with auxin.

## 1. Introduction

The innate static nature of plants is counterbalanced by their extraordinary ability to adapt their growth and development to the environment. The only possible way to endure environmental changes is to modify cell metabolism in response to abiotic or biotic stimuli, such as light, temperature, water and nutrient availability, or pathogen attacks. This adaptive plasticity is mediated at the molecular level by cellular receptors that perceive the stimulus and initiate a signalling cascade resulting in genetic changes that give rise to phenotypic trait modifications [1,2]. Plant hormones, small chemical messengers also known as phytohormones or plant growth regulators, participate in these intricate signaling networks, as they regulate many physiological processes involved in plant growth. In fact, independently or by interacting with each other, small concentrations of these naturally occurring substances are enough to initiate, modulate, and control stem cell maintenance, cell differentiation, elongation, and/or division. Hence, the possibility of survival of the sessile organisms comes from the peculiar plasticity of the somatic plant cells to de-differentiate, and, afterwards, re-differentiate, according to the hormonal stimuli. Thus, plants are able to form lost and/or new organs after stresses such as wounding, flooding, and etiolation, which allow a plant development suitable to the changed environmental conditions [3,4,5,6,7]. In this scenario, the naturally occurring adventitious root formation plays an important role in the plant biology. New roots can arise from excised portions of the stem and/or leaves, giving rise to new plant individuals, with their own new root system, physically separated from the “mother” plant. This means a huge potential for plant survival that allows for vegetative reproduction [8]. Horticulturists and/or nurserymen took advantage of adventitious root initiation of cuttings to rapidly reproduce plants by conventional means, and, nowadays, by the in vitro tissue culture technique. However, in both cases, adventitious root formation also constitutes a real bottleneck of any clonal propagation programme for elite plants. Thus, also because of its commercial impact, researchers have begun to deeply investigate this physiological process at various levels: genetic, molecular, and cellular, and to search for new rooting treatments.

**Figure 1:**
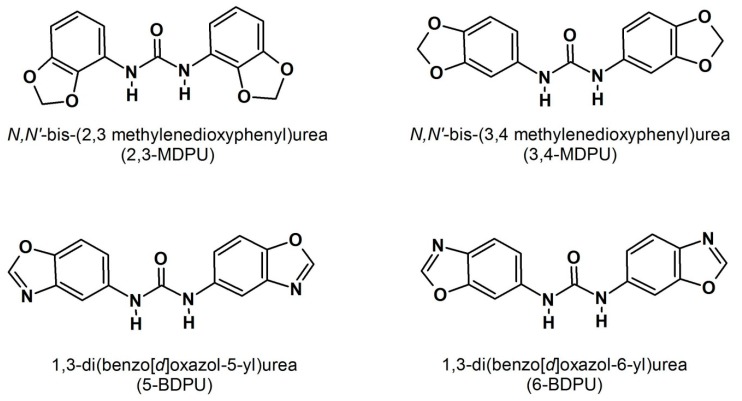
The molecular structure of urea derivatives.

Nowadays, it is widely known that the adventitious root formation is a postembryonic de novo organogenetic process by which the differentiated cell identity is reprogrammed to give rise to a root meristem where no roots were previously programmed [9,10]. Basic research carried out on model plants has shown that cells require hormones to acquire the competence to differentiate, primarily auxin, the key factor in stimulating the root formation, and secondly other plant growth regulators, with all of them participating in the complex regulation of the different phases of the process [11,12,13,14,15]. Moreover, it is not only a question of hormone cross-talks, but other internal factors (such as genetic traits and the metabolic and physiological status of the cutting) or environmental ones are critical to controlling the adventitious root formation [16,17,18,19,20]. On the other hand, a lot of applied research has been done to enhance adventitious rooting in difficult-to-root species, especially woody ones, by the utilization of bioactive compounds that could integrate or adjuvate the auxin activity. Cuttings have been supplemented with phenolic compounds [21], thiol compounds [22,23], amino acids [24,25], and carbohydrates [26,27] in combination with auxin to stimulate the root formation and to reduce its unfavourable side effects. In fact, it has been reported that adventitious roots may be stunted or malformed, as a result of the auxin treatment alone [28], giving rise to a poor-quality root system that will hardly support the ex vitro transfer and acclimatization. Starting from our structure–activity relationship studies, many years ago, we identified four symmetric urea derivatives, the *N*,*N*′-bis-(2,3-methylenedioxyphenyl)urea (2,3-MDPU), the *N*,*N*′-bis-(3,4-methylenedioxyphenyl)urea (3,4-MDPU), the 1,3-di(benzo[*d*]oxazol-5-yl)urea (5-BDPU), and the 1,3-di(benzo[*d*]oxazol-6-yl)urea (6-BDPU) (Figure 1), that enhance adventitious root formation without showing an auxin-like activity per se [29,30]. They were also totally ineffective when tested in specific cytokinin bioassays, which were performed as their chemical structure resembles that of some cytokinin-like urea derivatives [29,30]. Of the four urea derivatives, the 2,3- and the 3,4-MDPU were synthesized and studied first, and, therefore, they were considered to be lead compounds. Subsequently, the 5- and the 6-BDPU were synthesized by the replacement of the methylenedioxyphenyl group with the isostere benzoxazole. Their adjuvant rooting activity was characterized by being (*i*) closely dependent on the symmetric nature, as the asymmetric corresponding molecules were ineffective; (*ii*) independent of the simultaneous exogenous supplementation of auxin, or responsible for a reduced concentration of an exogenously supplemented auxin, depending on whether the species is easy- or difficult-to-root; (*iii*) untied from callus formation, as a consequence of the totally absent, or at least reduced, exogenous auxin supplementation; (*iv*) the 5- and the 6-BDPU, a little less effective than the lead compounds. We demonstrated that their considerably wide action spectrum includes herbaceous and woody Angiosperms as well as Gymnosperms, that their supplementation enhances adventitious rooting either in in vivo or in in vitro experimental conditions, and that this gives rise to a good-quality root system favoring the survival of rooted microcuttings [31]. Here, we summarize the recent results obtained about the positive effects of the supplementation of these urea derivatives, alone or in the simultaneous presence of auxin (Table 1), and we explain the state of the art about the nature of their interaction with auxin, the real adventitious root inducer.

## 2. Activities and Positive Side Effects of 2,3-MDPU/3,4-MDPU and 5-BDPU/6-BDPU Alone or in addition to Auxin

We enlarged the action spectrum of MDPUs, as we demonstrated that 2,3- and 3,4-MDPU could be positively used as adventitious rooting adjuvants in a micropropagation programme for *Capparis spinosa* L., a perennial shrub, as a clear example of spillover effect [32]. In fact, the importance of the caper production has greatly grown in the last few years, such as to its increased utilization either in food or in medicinal or cosmetic industries. This has required the production, by in vitro micropropagation, of a huge amount of elite plants, characterized by a good quality and genetic stability. To overcome the bottleneck of these large-scale vegetative production systems, i.e., the adventitious rooting of micropropagated cuttings, different rooting conditions, including supplementation with indole-3-acetic acid (IAA), indole-3-butyric acid (IBA), 1-naphthaleneacetic acid (NAA), and 2,3- and 3,4-MDPU, were evaluated at different concentrations. The best rooting result was achieved when microcuttings were cultured in the presence of 1 µM 2,3-MDPU, considering the percentage of rooted microcuttings (93/7%), the morphological appearance of a well-developed root system with a lot of lateral roots, the absence of callus formation, and the highest percentage of rooted microcuttings acclimatized to ex vitro conditions (82%). Last but not least, this treatment also resulted in the simplest in vitro culture practice, as the microcuttings were cultured in the light, in the continuous presence of the 2,3-MDPU, thus avoiding the need for a darkness period and the transfer under hormone-free culture conditions. Another piece of knowledge about the utilization of MDPUs as rooting adjuvants has been very recently achieved by Vielba and colleagues [33], who had grown *Castanea sativa* Mill. (chestnut) and *Quercus robur* L. (oak) microshoots in vitro in the presence of different concentrations of 2,3- and 3,4-MDPU plus an exogenous auxin. The researchers demonstrated that, in the presence of MDPUs, the rooting percentage was ameliorated, although not significantly, the percentage of rooted shoots that formed callus was reduced, and the root system showed a more elaborated architecture, with the development of a lot of lateral roots. Thus, these positive effects promoting plant anchorage, water uptake, and nutrient acquisition also resulted in a reduction of shoot tip necrosis in microshoots that looked taller and healthier than the pale ones obtained by the auxin supplementation alone. The results seemed to be species-specific and MDPU and auxin concentration-dependent. The histological study carried on in parallel to evaluate the activity of MDPUs in initiating roots at the shoot base revealed that the cell division appeared to occur earlier in root initials, thus confirming that the root system architecture seems to be modulated in the presence of these urea derivatives [33].

On the other hand, we more deeply investigated the involvement of 5-BDPU and 6-BDPU in the adventitious rooting process [34]. We demonstrated that BDPUs enhance adventitious root formation in distantly-related herbaceous and woody plants, namely *Arabidopsis thaliana* L. seedlings and *Pinus radiata* D. Don hypocotyl cuttings, in the absence or in the simultaneous presence of an exogenous auxin, respectively. Secondly, we reported for the first time that the enhancement effect obtained by BDPUs in the simultaneous presence of an exogenously added auxin is unrelated to the type of auxin, and that the BDPU concentration needs to be modulated, depending on the auxin strength. In fact, when IBA is added, high BDPU concentrations are needed to obtain adventitious rooting enhancement, while in the presence of NAA, a stronger auxin than IBA, a low concentration of BDPUs is enough.

Besides this, an empirical auxin quantification and localization was obtained by the in vitro culture of *Arabidopsis thaliana DR5::GUS* transgenic plants, which are sensitive to auxin in a dose-dependent manner. Thus, IBA plus 5-BDPU and NAA plus 5-BDPU induced a magnification of the β-glucuronidase (GUS) expression, as to the intensity and the extension of the staining, compared with that induced by the same concentration of IBA or NAA alone, respectively.

Another spillover effect was reached by rooting experiments with in vitro micropropagated cuttings of *Ceratonia siliqua* L. (carob tree) [35]. The main economic importance of this Mediterranean woody species is the seed production as a chocolate substitute, which is useful as a “functional food” for celiacs, diabetics, and for those intolerant to caffeine, or for gum extraction. Carob trees are also used for landscape architecture and for the reforestation of degraded areas, as they are tolerant to abiotic stresses, such as drought, salinity, and high temperature. However, large-scale propagation programmes are made difficult by the recalcitrance to adventitious rooting that some valuable genotypes show. Thus, carob microcuttings were cultured in vitro in the simultaneous presence of different concentrations of IBA plus 5- or 6-BDPU, for 3 days in the dark, and then transferred to a hormone-free medium in the light, for another 25 days. The percentage of rooted microcuttings was generally ameliorated by the presence of BDPUs and it was significantly enhanced by the treatment with IBA plus 5-BDPU, compared with IBA alone. Adventitious roots always emerged from the base of the cuttings, where callus formation was reduced or sometimes even absent (as already demonstrated by other experiments). Furthermore, from the histological study carried out in carob and *P. radiata* hypocotyl microcuttings, as representatives of Angiosperms and Gymnosperms, respectively, it was also reported that 5-BDPU alone enhances xylogenesis in both of these distantly related species, as an alternative programme to adventitious rooting, while, as a consequence of the simultaneous application of IBA and 5-BDPU, the adventitious root primordia were widely present [35].

## 3. Signaling Pathway of the Interaction between 2,3-MDPU/3,4-MDPU or 5-BDPU/6-BDPU and Auxin

Auxin has emerged as the real adventitious root formation stimulator, and is absolutely required to induce this developmental process *in planta* as well as in in vitro culture systems. Thus, MDPUs and BDPUs favour the adventitious rooting process only in the simultaneous presence of auxin, regardless of whether it is endogenous or exogenously added, and, in this latter case, as a natural or a synthetic hormone. All of the experimental data collected over the years suggest that MDPUs and BDPUs magnify the response to the auxin stimulus. In fact, the auxin concentration necessary to induce adventitious rooting of competent-to-root cuttings is lowered in the presence of these urea derivatives, thus reducing the amount of basal callus. Different approaches have been adopted to try to define the mode of action of these urea derivatives. Their possible interaction with auxin signaling pathways was investigated by analyzing the expression levels of either *P. radiata SCARECROW-LIKE1* gene (*PrSCL1*) or *P. radiata SHORTROOT* gene (*PrSHR*), induced in the cambial and rooting-competent cells of *P. radiata* hypocotyls, during the earliest stages of the adventitious root formation, before the resumption of the cell division and the appearance of the adventitious root primordia [36,37]. In this experimental system, the induction of *PrSHR* is not dependent on the presence of an exogenous auxin, while *PrSCL1* is in the auxin-dependent signalling pathway, resulting in the adventitious root formation, as it is induced in the presence of the optimal concentration of the exogenously added auxin (10 µM). Thus, we thought that the differential regulation of the induction of these genes could provide the opportunity to analyze the effects of MDPUs and BDPUs at the molecular level during the early stages of adventitious root formation. In the simultaneous presence of a low auxin concentration and of MDPUs or BDPUs, the expression of *PrSHR* was found to not be significantly affected, while the mRNA levels of *PrSCL1* were enhanced, in comparison with the mRNA levels of the same low auxin concentration [34,38]. Thus, we hypothesized that MDPUs and BDPUs could interact with the auxin signaling pathway before the activation of cell divisions.

Moreover, once again in the hypocotyl cuttings of *P. radiata* seedlings, the spatial distribution of endogenous IAA was investigated, trying to verify if 2,3-MDPU and 5-BDPU, as representatives of MDPUs and BDPUs, respectively, affect the local auxin pools at the basal wounded site of the cuttings. Localized IAA signals were observed in specific clusters of cells after 6 days of culture in the simultaneous presence of an exogenous auxin plus 2,3-MDPU or 5-BDPU, only. We tentatively explained this result through a series of modifications that could occur individually or together, at the level of specialized auxin-sensitive cells. In fact, the compounds may generally favour the interaction with local and/or long-distance auxin transport that originates in the auxin maxima. More precisely, they could be responsible for the localization of the auxin, or for the prevention of its dispersion or for a different sensitivity of cells to respond to the auxin stimulus. Whatever the reason, they allow for root meristem organization in the presence of a low exogenous auxin concentration, thereby reducing the callus formation. Similarly, the results obtained by *DR5::GUS* transgenic plants can also be explained by hypothesizing that 5-BDPU could affect the long-distance auxin transport or the auxin influx, or modify the cell sensitivity to the auxin [34].

Besides this, from the development of a high number of lateral roots, compared to that of the control conditions, detected in caper cuttings, in chestnut and oak microshoots supplemented with MDPUs and/or BDPUs, we can also infer that these urea derivatives have a putative positive effect on the auxin transport in the new roots, thereby promoting the local auxin accumulation from which new lateral root meristems originate.

## 4. Conclusions and Perspectives

This review reports the updated results regarding the effects of MDPUs and BDPUs on the adventitious rooting process. Their activity has been studied in different experimental systems, in distantly-related Angiosperms and/or Gymnosperms, in herbaceous and woody plants, in model plants, as well as in economically important ones. It has been demonstrated that MDPUs and BDPUs act as adventitious rooting adjuvant compounds in the simultaneous presence of auxin, the well-known plant growth regulator on which this organogenetic process depends. In fact, the number of rooted cuttings and the mean root number per rooted cutting are stimulated by their supplementation (although not always significantly). A reduction of the callus mass is obtained, and a lot of lateral roots are formed in the newly adventitious ones.

In the near future, further investigations will be carried out to evaluate the possible effect of MDPUs and BDPUs on the activity of the IAA influx carrier AUX1. This will also provide us with the opportunity to better understand if, and how, these compounds could affect the switching between the xylogenesis and the adventitious root formation. Moreover, with their urea-type chemical structure, it would be interesting to verify if MDPUs and BDPUs could directly or indirectly interfere with the cytokinin signaling pathway. In fact, it has been demonstrated that cytokinins have a promoting effect in the early induction phase of adventitious rooting during cell divisions, while they inhibit the process in the late induction phase. Thus, studying the MDPUs’ and BDPUs’ behaviour could be extremely important to elucidating the dynamic auxin–cytokinin crosstalk that regulates the adventitious root formation.

## Figures and Tables

**Table 1:** Summary of the most evident morphological effects of 2,3-MDPU, 3,4-MDPU, 5-BDPU or 6-BDPU supplementation.

Species	Molecule	Concentration	Effects	Ref.
*Malus pumila*	2,3-MDPU 3,4-MDPU 5-BDPU	1 μM 8, 16 μM	Microcuttings Enhancement of adventitious rooting	[29] [30]
*Vigna radiata*	5-BDPU 6-BDPU	1 μM	Shoots Enhancement of adventitious rooting	[30]
*Capparis spinosa*	2,3-MDPU 3,4-MDPU	1 μM 10 μM	Shoots Enhancement of adventitious rooting Enhancement of lateral root formation Absence of callus formation Enhanced acclimatization to ex vitro conditions	[32]
*Quercus robur*	2,3-MDPU 3,4-MDPU	1 μM (plus auxin)	Microshoots Enhancement of adventitious rooting Enhancement of lateral root formation Reduction of callus formation Reduction of shoot tip necrosis	[33]
*Castanea sativa*	2,3-MDPU 3,4-MDPU	1, 10 μM (plus auxin)	Microshoots Enhancement of adventitious rooting Enhancement of lateral root formation Reduction of callus formation Reduction of shoot tip necrosis	[33]
*Arabidopsis thaliana*	5-BDPU 6-BDPU	8 μM	Seedlings Enhancement of adventitious rooting	[34]
*Pinus radiata*	5-BDPU 6-BDPU	10 μM (plus auxins)	Hypocotyl cuttings Enhancement of adventitious rooting	[34]
*Ceratonia siliqua*	5-BDPU	10 μM (plus auxin) 10 μM	Microcuttings Enhancement of adventitious rooting Enhancement of xylogenesis	[35]
*Pinus radiata*	5-BDPU	10 μM	Hypocotyl cuttings Enhancement of xylogenesis	[35]
